# Investigating adult age differences in real-life empathy, prosociality, and well-being using experience sampling

**DOI:** 10.1038/s41598-022-06620-x

**Published:** 2022-03-02

**Authors:** Lena Pollerhoff, Julia Stietz, Gregory John Depow, Michael Inzlicht, Philipp Kanske, Shu-Chen Li, Andrea M. F. Reiter

**Affiliations:** 1 Lifespan Developmental Neuroscience, Faculty of Psychology, Technische Universität Dresden, Dresden, Germany; 2 Clinical Psychology and Behavioral Neuroscience, Faculty of Psychology, Technische Universität Dresden, Dresden, Germany; 3 Department of Psychology, University of Toronto, Toronto, ON Canada; 4 Rotman School of Management, University of Toronto, Toronto, ON Canada; 5 Max Planck Institute for Human Cognitive and Brain Sciences, Leipzig, Germany; 6 Centre for Tactile Internet with Human-in-the-Loop, Technische Universität Dresden, Dresden, Germany; 7 Department of Child and Adolescent Psychiatry, Psychosomatics and Psychotherapy, University Hospital Würzburg, Würzburg, Germany; 8 German Centre of Prevention Research On Mental Health, Julius-Maximilians-Universität Würzburg, Würzburg, Germany

**Keywords:** Human behaviour, Ageing

## Abstract

While the importance of social affect and cognition is indisputable throughout the adult lifespan, findings of how empathy and prosociality develop and interact across adulthood are mixed and real-life data are scarce. Research using ecological momentary assessment recently demonstrated that adults commonly experience empathy in daily life. Furthermore, experiencing empathy was linked to higher prosocial behavior and subjective well-being. However, to date, it is not clear whether there are adult age differences in daily empathy and daily prosociality and whether age moderates the relationship between empathy and prosociality across adulthood. Here we analyzed experience-sampling data collected from participants across the adult lifespan to study age effects on empathy, prosocial behavior, and well-being under real-life circumstances. Linear and quadratic age effects were found for the experience of empathy, with increased empathy across the three younger age groups (18 to 45 years) and a slight decrease in the oldest group (55 years and older). Neither prosocial behavior nor well-being showed significant age-related differences. We discuss these findings with respect to (partially discrepant) results derived from lab-based and traditional survey studies. We conclude that studies linking in-lab experiments with real-life experience-sampling may be a promising venue for future lifespan studies.

## Introduction

Throughout the lifespan, satisfying social interactions are key for well-being (e.g.,^1^) as well as for mental and physical health (e.g.,^2^). Experiencing empathy in response to someone’s suffering, a feeling that is believed to trigger prosocial behavior, is an important ingredient for establishing and maintaining relationships with other people^3,4^. Whilst it is certainly indisputable that social functioning remains important throughout the adult lifespan^5,6^, findings on how empathy and prosociality develop and interact over the course of adulthood are still mixed.

Research on the lifespan development of empathy is often built on the distinction of affective components (affect sharing, empathic concern, compassion) from cognitive components (perspective taking) of empathy^7,8^. In two recent laboratory studies using a naturalistic paradigm to dissociate both affective and cognitive aspects of understanding others^9,10^, we did not observe age-related differences in affect sharing. However, and in line with our expectations, empathic concern was found to be enhanced whereas perspective taking ability decreased in older compared to younger adults. Whilst this pattern of findings contributes to an emerging prevalent view of reduced cognitive but preserved or increased affective empathy in older vs. younger adults (^8^ for a recent review), there is substantial heterogeneity in the literature, particularly when considering adult age effects across the lifespan.

With respect to experimental studies, there is some evidence for an age-related increase in task-based (i.e., less naturalistic behavioral measure conducted in the lab) empathic concern^11^, and affect sharing^12^ in some studies. However, other comparable studies find no evidence for significant age-related differences with respect to task-based empathic concern^13,14^, and affect sharing^15^. The same inconsistencies are observed in terms of perspective taking when measuring the construct in behavioral tasks. Overall, meta-analytic evidence points towards a decrease of perspective taking ability^16^ but, there are other experimental studies showing no evidence for age differences in perspective taking^17–20^. With respect to studies that measure empathic concern, affect sharing, or perspective taking with self-report, results have suggested linear and inversed U-shaped relationships between age and self-reported empathic concern^11,21^ and perspective taking^21^, as well as age-related declines in self-reported affect sharing^22,23^.

The understanding of developmental change and stability with respect to prosociality has become an important research topic^24,25^. Similar to the heterogeneity of findings in the domain of empathy, previous research on adult age differences in prosocial behavior has yielded mixed results. Experimental age-comparative laboratory studies have often revealed a higher degree of prosocial behavior in older compared to younger adults^14,26–29^, and have shown a linear increase in prosociality when examined across the adult lifespan^11,30,31^. There are, however, other studies which do not find such age-related increases in prosocial behaviors neither in experimental tasks or self-reported prosocial measures when comparing younger and older adults^32,33^, when comparing middle- and older age groups^34^, nor when looking at age correlations across adulthood^35,36^.

Social psychology and neuroscience research suggests that feeling empathy with a person usually results in greater prosocial behavior (for reviews see^37–40^). Some previous studies have asked whether this link might be moderated by adult. Experimental studies in the laboratory demonstrate enhanced prosocial behavior after an empathy induction in older adults (compared to younger adults)^14^ as well as age-related linear increases in prosocial behavior across younger, middle aged, and older adults that were partially mediated by empathic concern^11^. Age was also found to positively moderate the association of self-reported prosocial behavior and empathic concern, but only in participants younger than 75 years^34^. However, another study did not find age-related differences regarding the link between empathy and prosocial behavior when comparing younger and older adults^27^.

The question of whether subjective well-being, used as an umbrella term for feelings of happiness, a sense of purpose in life, and life satisfaction^2^, changes over the course of the adult lifespan is a research topic that has also attracted much attention over the last decades (e.g.,^41,42^). Most prominently, an influential lifespan theory of affective development (‘socio-emotional selectivity theory’^43^) postulates higher socio-emotional well-being in older adults. It is argued that when individuals perceive their remaining lifetime as limited, they tend to prioritize present goals like optimizing socio-emotional well-being^44^. This is thought to be related to a “positivity effect”, i.e., a motivational shift to positive over negative information processing^45–47^, even though there are studies that do not find evidence for age-related differences regarding this processing bias for positive stimuli^48,49^. Large-scale international surveys across several countries often reveal a U-shaped association between age and evaluative well-being, illustrating higher well-being in younger and older adult age (e.g.,^50,51^). Studies using ecological momentary assessment or other daily measures also show a U-shaped relationship of adult age and life satisfaction^52^, or a curvilinear relationship of age and negative emotional experience^53^. Notwithstanding, there is a current debate about the putative U-shaped pattern, including critiques with respect to its robustness and generalization^54–56^. Prosocial behavior and well-being have been suggested to be associated with each other, and some studies have suggested that their link is moderated by age. In a recently published large-scale daily diary study^57^, younger adults’ prosocial behavior was associated with both costs and benefits, in that they experienced both greater negative affect and more positive experiences at the same time, while these associations were both attenuated in older adults. This was interpreted as a decreasing influence of prosociality on well-being over the course of the life span. In contrast, earlier age-comparative studies have pointed towards more beneficial effects of volunteering on life satisfaction in older compared to younger adults^58^.

Considering the lifespan developmental findings (and their considerable heterogeneity) reviewed above, an intriguing open research question is whether there are adult age differences in empathy, prosociality, and well-being as they occur in daily life. Ecological momentary assessment (EMA) is a method which is well-suited to capture these processes in real life. EMA is a relatively new method, with advantages like increased ecological validity, and decreased recall bias, while measuring within-person variability and change over a short time period^59^. These advantages may be particularly important for lifespan developmental studies, as recall bias^60^ and within intra-individual variability^61^ have been shown to be subject to age effects in different behavioral contexts. Unfortunately, and to the cost of external validity, to date only a small list of studies^4,62,63^ examined empathy and prosociality using EMA, most of them with no focus on adult age differences. With the current study we aimed to fill this gap by leveraging the advantages of EMA and investigating daily empathy, prosociality, and well-being under real-life circumstances, repeatedly per day within person. To this end we analyzed smartphone based experience-sampling data recently published by Depow and colleagues^63^. The primary goal of Depow and colleagues’ study^63^ was to analyze the perception of empathy in everyday lives and the prediction of prosocial behavior and subjective well-being (defined as feelings of happiness and purpose of life). They used quota-sampling to ensure their sample was representative of the U.S. adult population on key demographics, including age. However, age effects were not part of their original analysis. Given the growing interest in age-related differences regarding components of the social mind^24^ and the lack of real-life data in this field, we used the data acquired and provided openly by Depow et al.^63^ to examine age effects with regards to daily empathy, prosocial behavior, and well-being. Separating contributions of within- from between-subject variability, we aimed to investigate whether age influences daily empathy, prosocial behavior, and well-being, as well as their interactions. Of note, in the current study, empathy was defined as an umbrella term, subsuming the three different subcomponents emotion sharing, compassion, and perspective taking. Prosocial behavior was defined as an opportunity to help someone.

Based on the heterogeneity of age-related findings regarding the constructs of empathy and prosocial behavior as reviewed above, and considering inconsistencies in the definition of empathy, we had undirected hypotheses in terms of age differences in daily empathy and prosocial behavior. In line with previous results^50–52^, we expected a U-shaped relationship of adult age with well-being (measured in terms of happiness and sense of purpose), with higher well-being in younger and older adults. Based on a positivity bias postulated for older adults in the lifespan theories of emotional aging^45–47^, we expected older adults to experience more empathy in contexts with positive valence (i.e., situations where the target emotion was positive). Based on a previous lab-based study^34^, we hypothesized an attenuated relationship of empathy and prosocial behavior in the older age range. Furthermore, based on the literature^57^ we assumed a greater relationship of empathy and well-being with increasing age and expected the same age-related pattern with respect to the link between prosocial behavior and well-being.

## Results

**Figure 1:**
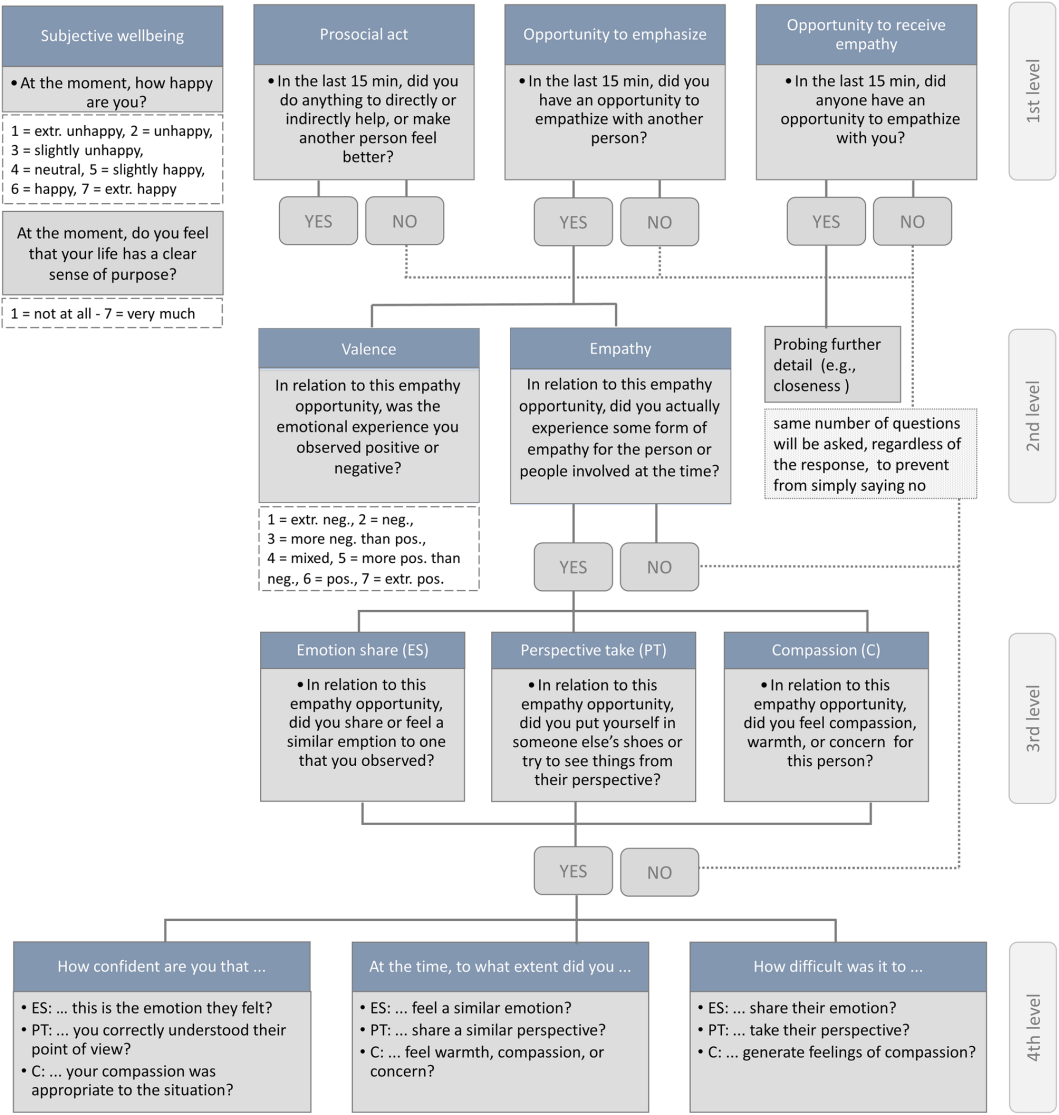
Daily survey design, visualizing the different survey levels and related questions. Only the questions relevant for the current study are depicted here. For further details, and a full study protocol, see Depow and colleagues^63^. Note that empathy (level 2) was assessed as an umbrella term, thus, questions about the subcomponents (level 3) emotion share, perspective taking, and compassion were only rated if the participant indicated to have actual feelings of empathy on level 2 of the survey. For each reported subcomponent on level 3, participants were asked about confidence, extent, and difficulty on level 4 of the survey.

In the current study we reanalyzed open-source data from Depow and colleagues^63^, using EMA in a demographically representative sample across the adult lifespan (final sample *n* = 243, 136 females, 104 males, and 3 others) to measure daily empathy, prosociality, and well-being. In the main experience sampling survey participants underwent seven surveys per day for one week, including four levels, building on each other (see Fig. 1). The first level measured daily well-being and prosocial behavior, as well as opportunities to empathize with someone or any situations where the participant could be the target of empathy. In case they had reported an empathy opportunity before, on the next (2nd) level participants answered questions about their actual feelings of empathy. On the third level the subcategories emotion sharing, perspective taking, and compassion were investigated. On the fourth level the extent, difficulty, and confidence for every indicated subcomponent were probed. Depow and colleagues^63^ collected information on participants’ age by asking about participants’ age group; participants did not provide information on their exact age. Age was binned into four ordered age groups, based on the original classification from Depow and colleagues^63^: (1) 18 to 34 years (*n* = 71, 45 females, 3 “other), (2) 35 to 44 years (*n* = 59, 28 female), (3) 45 to 54 years (*n* = 51, 34 female), and (4) 55 years and older (*n* = 62, 29 female). Thus, age group entered all models as a continuous variable, based on the assumption of ordinality and continuity (e.g.,^11,64^). Outcome variables derived from all four levels of the survey (see Fig. 1) were analyzed. All outcome variables were analyzed by using two (generalized) mixed-effect models, one including age group as linear predictor and one including age group both as a linear (age group) and a quadratic predictor (age group^2^). We report p-values corrected based on the false-discovery rate.

### Adult age differences in everyday empathy

#### Empathy opportunities

Regarding the first level of the survey (see Fig. 1), i.e., the frequency of empathy opportunities, and the frequency of being the target of empathy, no age-related differences were found (empathy opportunities: *b* = 0.01, *SE* = 0.08, *z* = 0.16, *p* = 0.872, adj. *p* = 0.872, *r* = 0.00; target of empathy: *b* = 0.02, *SE* = 0.10, *z* = 0.21, *p* = 0.834, adj. *p* = 0.872, *r* = 0.01). Reassuringly, this null effect of age suggests that potential age differences in all subsequent analyses are unlikely to be affected by baseline differences in how often different age groups experienced an opportunity to empathize in the first place.

#### Experiencing empathy

**Figure 2:**
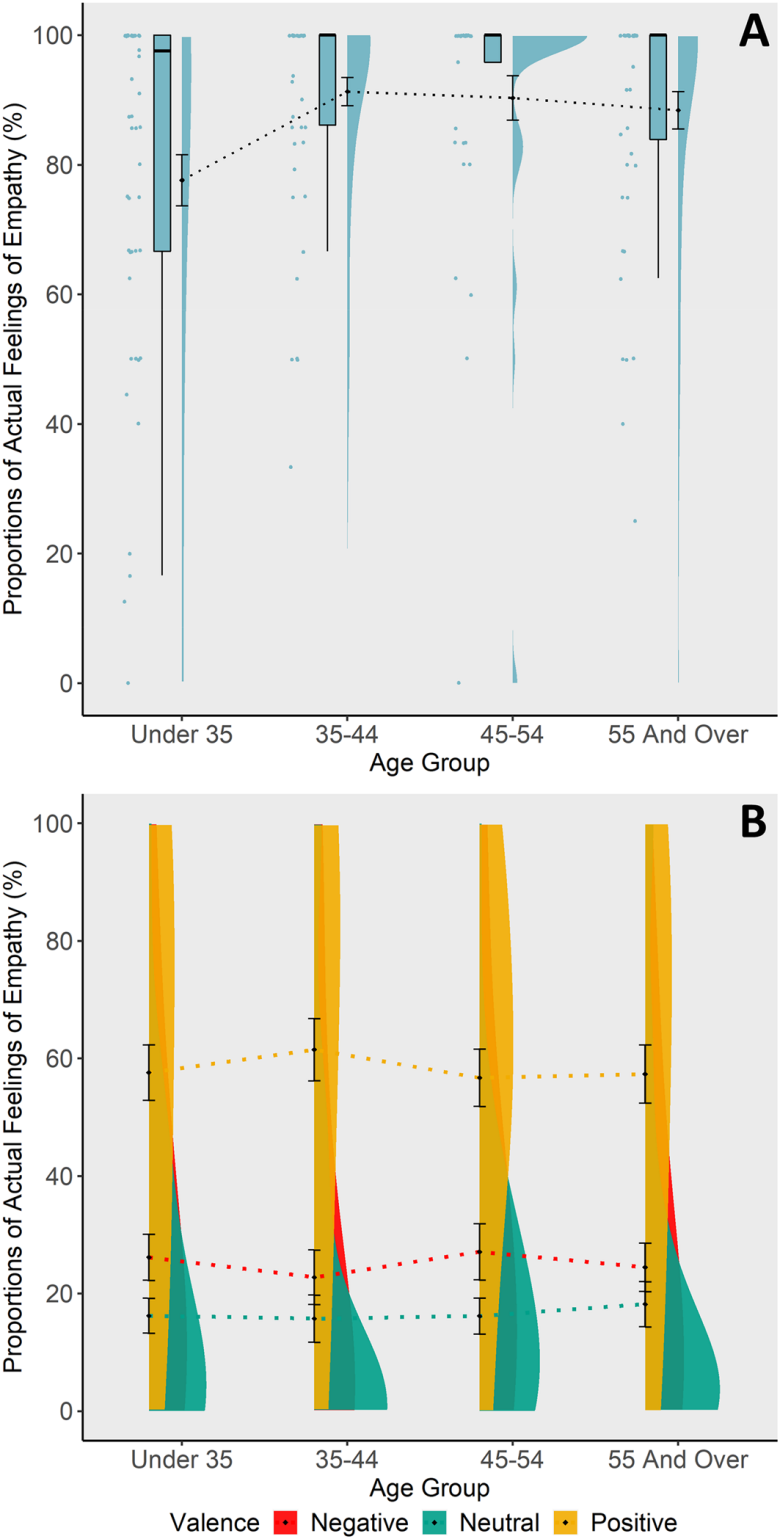
Daily empathy. The y-axis shows the proportions of answering ‘yes’ when asked about the actual feelings of empathy relative to the total amount of answered surveys. (**A**) Adult age differences regarding actual feelings of empathy. Significant quadratic association of actual feelings of empathy and age. (**B**) Interaction between valence (negative, neutral, or positive target emotion) of the empathy opportunity and age on reported feelings of daily empathy. Age did not moderate the link between the valence of the situation and actual feelings of empathy.

With respect to the actual feelings of empathy (2nd level of the survey), significant linear as well as quadratic effects of age were observed (linear: *b* = 0.37, *SE* = 0.15, *z* = 2.44, *p* = 0.015, *r* = 0.10; quadratic: *b* = -0.41, *SE* = 0.18, *z* = -2.24, *p* = 0.025, *r* = -0.11). Daily feelings of empathy increased across the first three age groups, from 18 to 44 years, but, as the significant quadratic trend suggests, show a tendency to decrease beyond these ages, in those 55 years and older (see Fig. 2A).

In lifespan developmental theories, a positivity bias for older as compared to younger adults has been suggested^45–47^. Here, we tested this hypothesis with respect to the role of emotional valence (i.e., positive, neutral, or negative) of the empathy opportunity on actual feelings of empathy. That is, we tested for an interaction of age group with valence of the empathy opportunity. As reported in Depow and colleagues^63^, subjects generally showed higher actual feelings of empathy after positive empathy opportunities; however, contrary to our expectation this effect was not moderated by age (Chi^2^ (2) = 0.27, *p* = 0.872, see Fig. 2B).

#### Subcategories of empathy

Regarding the subcomponents of empathy (3rd level of the survey, Fig. 1), no significant age-related differences (neither linear, nor quadratic) were found for emotion sharing (*b* = -0.17, *SE* = 0.13, *z* = -1.31, *p* = 0.189, adj. *p* = 0.567, *r* = -0.05), perspective taking (*b* = 0.01, *SE* = 0.14, *z* = 0.06, *p* = 0.952, adj. *p* = 0.952, *r* = 0.00), or compassion (*b* = 0.18, SE = 0.35, *z* = 0.52, *p* = 0.601, adj. *p* = 0.902, *r* = 0.05). Note that ratings of the subcomponents of empathy were only provided by those participants who indicated actual feelings of empathy on the second level of the survey. Thus, while we observe age-related differences in the tendency to experience actual feelings of empathy in general (2nd level), among the proportion of participants who experienced actual feelings of empathy, no age differences with regard to the subcomponents were observed. Further, after adjusting p-values, no age-related differences were observed in the extent, difficulty, and confidence (4th level) of the different subcomponents: emotion sharing, perspective taking, and compassion (all *b*s < 0.04, all *SE*s < 0.08, all *t*s < 0.96, all adj. *p*s > 0.081, all *r*s < 0.20; see supplementary table S1).

Depow and colleagues^63^ showed that the co-occurrence of the three subcomponents of empathy (i.e., emotion sharing, perspective taking, and compassion) was very high. Thus, the different components of empathy seem to mainly co-occur in everyday life. Analyzing age effects on the co-occurrence of the empathy subcomponents using a chi^2^-tests, we did not find any significant effect of age (all chi^2^s < 5.76, all *p*s > 0.124; see supplementary figures S1-4).

## Adult age differences in prosociality

**Figure 3:**
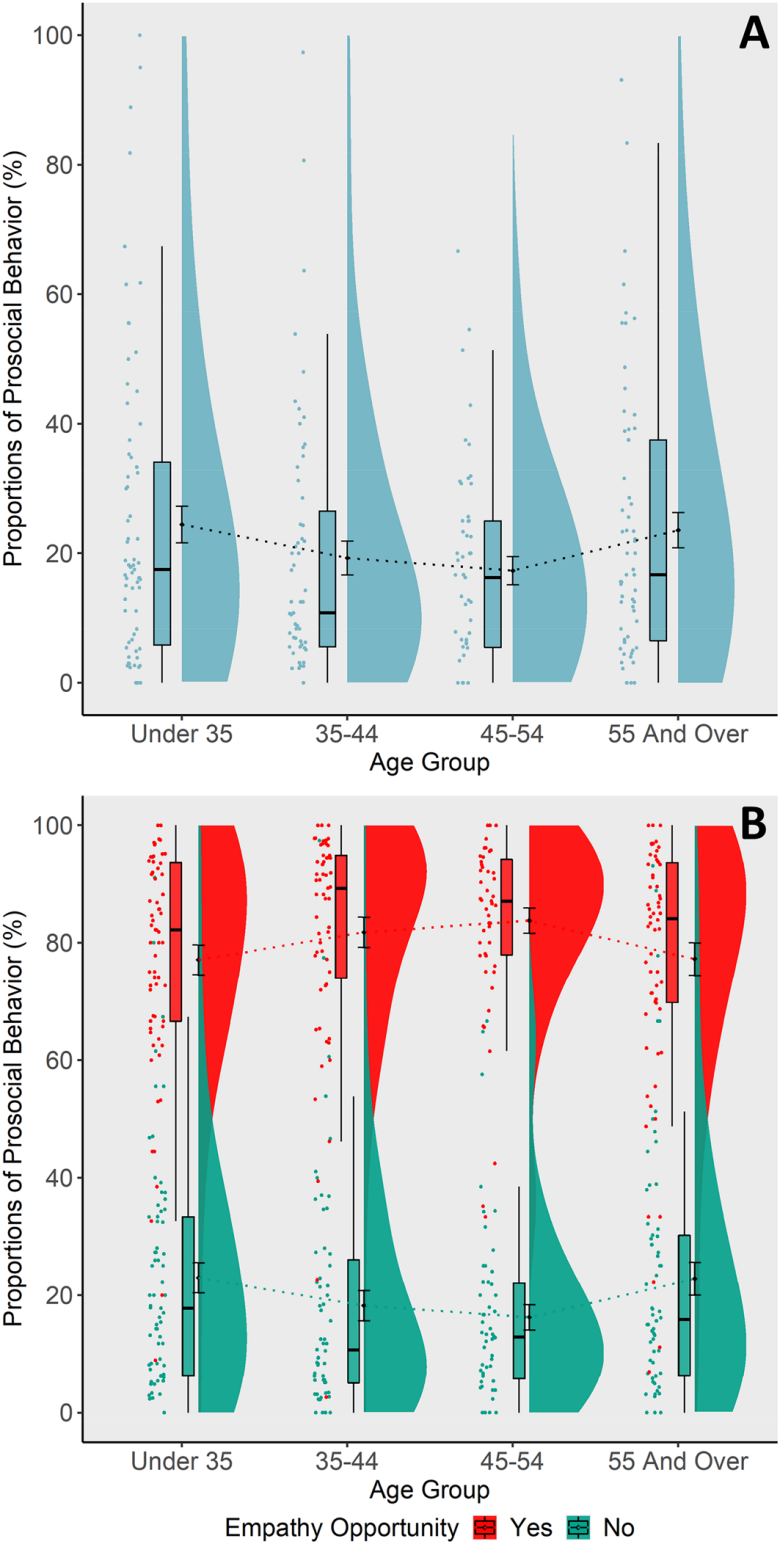
Daily prosocial behavior. The y-axis shows the proportions of answering ‘yes’ when asked about acting prosocially relative to the total amount of answered surveys. (**A**) Adult age differences in prosocial behavior. No significant association between age and prosocial behavior was found. (**B**) Within-subject effect of empathy opportunity x age on prosocial behavior. All age groups showed more prosocial behavior after an empathy opportunity. A quadratic effect of age group indicated that this effect was more pronounced in the middle-aged groups than in the younger and older age group.

Contrary to our expectations, we did not find significant age-related differences with respect to daily prosocial behavior (*b* = 0.007, *SE* = 0.08, *z* = 0.08, *p* = 0.934, *r* = 0.00, see Fig. 3A). Depow and colleagues^63^ found significant associations between different aspects of everyday empathy (e.g., empathy opportunity, actual feelings of empathy, subcomponents) and prosocial behavior. In the current analysis most of those effects were not significantly moderated by participants’ age (all adj. *p*s > 0.227, see Table 1). Only the interaction term of reported empathy opportunities and age as quadratic and linear term, respectively, significantly predicted prosocial behavior as a within-subject effect (linear: *b* = -0.13, *SE* = 0.05, *z* = -2.80, *p* = 0.005, adj. *p* = 0.024, *r* = -0.04, quadratic: *b* = 0.15, *SE* = 0.06, z = 2.72,* p* = 0.006, adj. *p* = 0.024, *r* = 0.04). Across all age groups the experience of an empathy opportunity was associated with higher prosocial behavior, but this effect was more pronounced in the middle-aged groups than in the youngest and oldest groups (see Fig. 3B).

**Table 1:** Within- and between person effects of different interactions regarding different aspects of daily empathy and age predicting daily prosocial behavior.

Interaction term predicting prosocial behavior	Within-subject effects				Between-subject effects			
	*t* or *z*-score	Adj. *p *value	Estimate (*SE*)	Effect size (*r*)	*t* or *z*-score	Adj. *p *value	Estimate (*SE*)	Effect size (*r*)
Empathy opportunity * age group	− 2.8	0.024*	0.05	− 0.04	− 1.9	0.406	0.2	− 0.1
Empathy opportunity *age group^2^	2.72	0.024*	0.06	0.04				
Target of empathy* age group	1.12	0.351	0.04	0.01	− 0.94	0.819	0.32	− 0.08
Empathy* age group	1.13	0.351	0.09	0.03	0.03	0.978	0.31	0
Emotion share* age group	− 1.43	0.308	0.11	− 0.04	0.81	0.819	0.33	0.07
Perspective take* age group	− 1.72	0.227	0.11	− 0.05	0.52	0.844	0.32	0.05
Compassion* age group	0.42	0.674	0.15	0.02	0.25	0.94	0.41	0.03

Interaction term predicting prosocial behavior	Chi^2^	Adj. *p*-value			Chi^2^	Adj. *p*-value		
Valence^α^*age group^β^	0.2	0.674			0.53	0.819		

Prosocial behavior was included in all models as binary outcome variable. Statistics obtained from mixed models, nested within participant and survey day. Each interaction ran in a separate model, with age as linear and quadratic term separately. Model selection was conducted based on a loglikelihood ratio test. *P *values were adjusted to control the false discovery rate. *p < 0.05.

^α^Positive, negative, or neutral target emotion.

^β^Reduced random effect structure due to convergence warnings, only nested within participant.

## Adult age differences in subjective well-being

**Figure 4:**
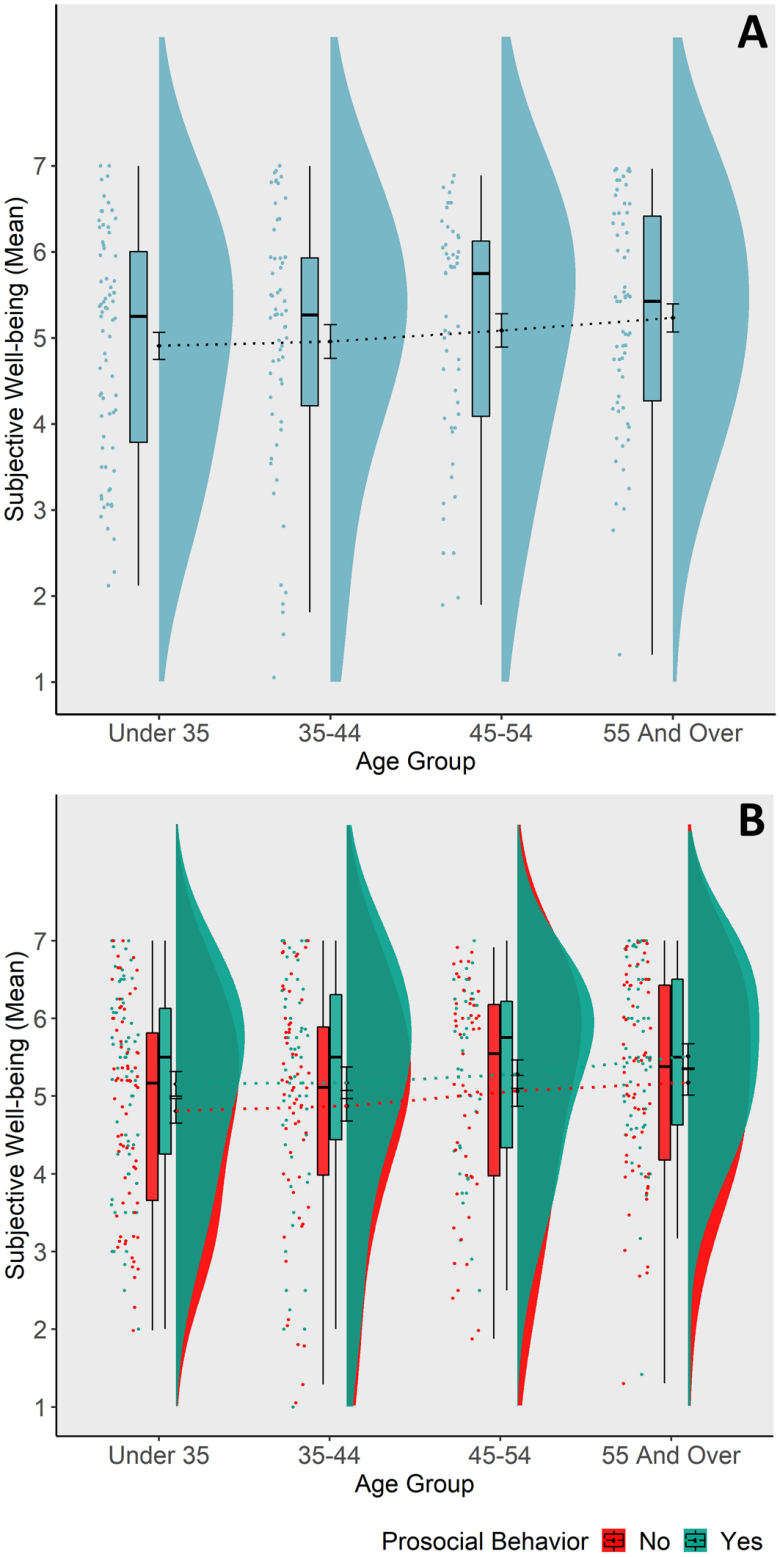
Daily well-being. (**A**) Adult age differences in subjective well-being. No significant association between age and well-being. (**B**) Within-subject effect of prosocial behavior x age on well-being. Positive association between well-being and acting prosocially, irrespective of participants’ age. Stronger association of a prosocial act with wellbeing in younger adults.

Overall, and contrary to our a-priori hypothesis, we did not find significant adult age effects on daily subjective well-being (*b* = 0.11, *SE* = 0.08, *t*(233) = 1.48, *p* = 0.141, *r* = 0.10, see Fig. 4A). In Depow and colleagues’^63^ analyses, different aspects of daily empathy (e.g., empathy opportunity, and actual feelings of empathy) were associated with subjective well-being. Thus, in a next step, we analyzed whether such an association of different aspects of daily empathy and well-being, as reported on the group-level, was moderated by age. No significant interaction with age was revealed (all adj. *p*s > 0.154, see Table 2). We found a significant interaction effect between age and prosocial behavior on subjective well-being, but only as a within-subject effect (*b* = 0.03, *SE* = 0.01, *t*(6315) = 2.62, *p* = 0.009, *r* = 0.03; see Table 2). As can be seen from Fig. 4B, within-person, across all age groups, higher well-being was associated with acting prosocially before. However, the difference in well-being as a function of an opportunity to act prosocially was slightly more pronounced in the younger age groups (see Fig. 4B).

**Table 2:** Within- and between person effects of different interactions regarding different aspects of daily empathy and age predicting daily well-being.

Interaction term predicting well-being	Within-subject effects				Between-subject effects			
	*t* or *z*-score	Adj. *p*-value	Estimate (*SE*)	Effect size (*r*)	*t* or *z*-score	Adj. *p*-value	Estimate (*SE*)	Effect size (*r*)
Empathy opportunity * age group ^α^	0.43	0.667	0.01	0.01	− 2.03	0.154	0.35	0.13
Target of empathy* age group ^α^	0.52	0.667	0.01	0.01	− 2.26	0.154	0.40	0.15
Empathy* age group^α^	0.74	0.643	0.04	0.02	1.30	0.341	0.30	0.09
Emotion share* age group	− 1.17	0.569	0.04	0.04	− 0.13	0.931	0.30	0.01
Perspective take* age group	− 1.35	0.569	0.04	0.04	1.02	0.435	0.31	0.07
Compassion * age group	− 0.96	0.593	0.05	0.03	− 0.09	0.931	0.39	0.01
Interaction term predicting well-being	*F*-value	Adj. *p*-value			*F*-value	Adj. *p*-value		
Valence^β^* age group	1.73	.569			2.86	.212		

Interaction term predicting well-being	*t* or z-score	*p*-value	Estimate (*SE*)	Effect size (*r*)	*t* or *z*-score	*p*-value	Estimate (*SE*)	Effect size (*r*)
Prosocial Act* age group	2.62	0.009**	0.01	0.03	− 1.29	0.198	0.34	0.08

Well-being was included in all models as continuous outcome variable. Statistics obtained from mixed models, nested within participant and survey day. Each interaction ran in a separate model, with age as linear and quadratic term separately. Model selection was conducted based on a loglikelihood ratio test. P-values were adjusted to control the false discovery rate. ** *p* < 0.01.

^α^ Between-subjects effect models include religiosity as covariate.

^β^ Positive, negative, or neutral target emotion.

## Discussion

In this study, we used experience sampling data^63^ to test adult age differences with respect to empathy, prosociality, and well-being. Based on a representative sample, we were able to yield several new insights into the lifespan development of these important social functions, as well as their interactions in daily life.

With respect to daily empathy, we found a (small) linear effect of age on empathy across the lifespan, which is in line with postulated stable or even increased socio-affective processes in older adults, as measured in laboratory studies (e.g.,^9–11^). Interestingly, in this ecologically valid description of a near-representative sample we found an inverted U-shaped relationship of age and empathy. This pattern suggests that daily empathy increased from 18 years on, with a peak in midlife, but decreased slightly in the oldest group (55 years and older). Notably, the empathy level of the oldest group was higher than the one of the youngest. This pattern is in line with the findings from O’Brien and colleagues^21^, with respect to their quadratic associations in self-reported trait empathic concern and perspective taking. Further, there is cross-sectional and longitudinal evidence that moving from early to middle adulthood is associated with more stable dispositional traits like agreeableness^65^ and that the period of middle adulthood is also linked to highest values of generativity^66^, potentially rendering empathy a particularly relevant skill in this period of life.

When comparing the results of this experience-sampling study to previous research on the lifespan development of empathy, it is important to note that the definition of the construct empathy varies across studies, which has been criticized recently^67^ reminiscent to a discussion about so-called “Jingle-Jangle fallacies”^68,69^. Such an inconsistency in the definition of empathy also affects studies investigating age differences in empathy. In the current study, empathy was regarded as an umbrella term spanning the subcomponents emotion sharing, compassion, and perspective taking; consequently, participants only provided ratings for these subcomponents when they had indicated feelings of empathy before. Based on this operationalization, a high co-occurrence between emotion sharing, perspective taking, and compassion was reported, and no age-related differences regarding the three subcomponents nor their co-occurrence were found. Previous ageing studies differentiated (emotional) empathy in terms of affect sharing, from compassion, and perspective taking (e.g.,^9,10^), by assessing them independently from each other, which revealed differential age-related findings for each construct (^7,8^ for review).

The majority of studies on empathy thus far focused, by design, on empathy exclusively in the context of negative stimuli and most often with strangers in a lab (e.g., EmpaToM-Paradigm^70^, as used in^9^). A commendable recent exception by Ziaei and colleagues^71^ included positive and negative stimuli when measuring empathy in a behavioral task in the lab, demonstrated that older adults responded significantly slower to negative than to positive stimuli. Interpreting this result, the authors argued there is a greater difficulty in processing negative emotions in older adults. The dataset used for the current analyses offered the opportunity to examine age effects in interaction with the valence of a real-life situation. Indeed, Depow and colleagues^63^ showed that empathy was reported more frequently following situations with a positive valence. In contrast to a hypothesized positivity bias^71^, this effect was not significantly enhanced with increasing age. This is in line with a previous study conducted in the lab^48^ which could not find behavioral age-related differences in working memory performance as a function of emotional valence. However, the experience of affective empathy, particularly compassion^72^, be it in an emotionally positive or negative context, might be rewarding in itself or subsequently lead to satisfying social interactions (if, e.g., followed by prosocial behavior as demonstrated in the current dataset). In this regard, experiencing enhanced empathy in both positive and negative domains is in line with the lifespan goals suggested by emotional selectivity theory^43^, namely an enhanced pursuit of emotionally satisfying interactions in midlife and older age.

In this experience-sampling study, no adult age differences in prosocial behavior were observed. This is in contrast to most laboratory studies using economic decision-making tasks (e.g., dictator game, public good game), or experimental helping/donating paradigms, which showed pronounced prosocial behavior in older adults (e.g.,^14,26,73^). Also studies using self-report measures in terms of validated questionnaires, showed greater prosociality with advancing age, by comparing younger vs. older adults^74^, across younger and middle-old adults^75^, or across the whole adult lifespan^76,77^. While these inconsistencies in results may be argued to be due to these studies not measuring behavior in real life, discrepancies also occurred when comparing the current results with studies that measured real-life prosociality. Cavallini et al.^34^ reported a significant negative association between age and self-reported real life prosocial activities, arguing that real-life prosocial behavior is more cognitively and physically demanding. An important difference between the study by Cavallini and colleagues^34^ and the current study is the higher age on average, as they only included participants aged 55 years and older, but no younger age group. Thus, our comparably young sample here might not be ideally suited to detect potential effects of physical and cognitive demands on an aging effect in prosocial behavior. Further, in Cavallini and colleagues’ study^34^ prosocial behavior was not measured using EMA (e.g., in the last 15 min), but based on a self-reported questionnaire which asked about the recalled frequency of acting prosocially in a pre-defined set of prosocial scenarios during the last 12 months. It is possible different types of prosocial acts are assessed by self-reported questionnaires compared to EMA measures. Cavallini et al.’s^34^ method has potentially captured acts that are more memorable over a longer time frame and thus might have been more resource-intense (e.g., charitable giving). In the current study, prosociality is defined more broadly (as anything including direct or indirect help, or to make another person feel better). Thus, daily acts (including smaller acts) of prosociality (e.g., holding the door open) would be included in the EMA-based self-report. Consequently, it is plausible that EMA-based self-report captures acts that might have been forgotten otherwise. Due to such memory effects, or recall biases, which are more susceptible in global retrospective reports^78^, a difference in the reference time frame of self-report (12 months in Cavallini et al.’s study^34^ vs. 15 min in the current study) could be particularly relevant for lifespan studies, as there is evidence for an increased memory-experience gap in older individuals^60^.

We expected an attenuated relationship of empathy and prosocial behavior with higher age^34^ which was only partially confirmed. Age did not modulate the associations between actual feelings of empathy, nor of any of its subcomponents with prosocial behavior. However, the interaction between the opportunity to empathize, and age, both as a linear and a quadratic term on prosocial behavior was statistically significant as a within-person effect. Meaning all age groups showed more prosocial behavior when there was an empathy opportunity. However, this stronger tendency to act prosocially if there was a situation eliciting empathy (as compared to when there was none) was found to be slightly more pronounced in the middle-aged group of the sample. This is different compared to the results by Cavallini and colleagues^34^, which showed that with increasing age (> 75 years), daily prosocial behavior was less driven by empathic concern. When comparing these results. When comparing these results it should be noted the participants in Cavallini and colleagues^34^ were considerably older, and the self-reported measure reflected memories of prosocial acts within the last 12 months. Taken together, it leaves an open research question on what drives prosocial behavior in older adults’ everyday life.

In the current study, we did not observe the often-found U-shaped age-related pattern of self-reported well-being previously found in larger studies (e.g.,^50,52^). Subjective well-being was assessed by two different questions, merged into one universal well-being score, covering hedonic well-being (i.e., experienced happiness) and eudaimonic well-being (i.e., purpose of life). Even though eudaimonic well-being gets more attention in the current literature^2,79^, most studies that have found a U-shaped pattern of subjective well-being emphasized a hedonic operationalization of well-being (e.g.,^51,54^). However, beyond the measurement level, it is noteworthy to discuss the recent debate about the robustness and generalization of the putative U-shaped pattern with regard to the association of adult age and well-being^54–56^. A recently published study^54^ challenged this often as a typically assumed U-shaped pattern, by revealing inconsistent findings with respect to the association of age and well-being, both in cross-sectional and longitudinal studies. Additionally, it is argued that age effects on well-being could be an epiphenomenon of other variables, reflecting the current life circumstances associated with ageing (e.g., income, education), fixed effects like personality traits, or selection effects which might differ as a function of age group^51,56,80^.

We confirmed a hypothesized moderation effect of age on the link between daily prosocial behavior and subjective well-being. Across all age groups, subjective well-being within a person was higher when a prosocial act had been was performed 15 min before. However, this trend was attenuated with increasing age. This is in line with Chi and colleagues’^57^ recently published observations about a decreased influence of prosocial behavior on well-being in older compared to younger adults. Interestingly, an earlier study^58^ observed the opposite when looking at the long-term impact (i.e., over 3 years of time) of volunteering in a longitudinal survey, namely enhanced well-being as a result of volunteering in older compared to younger adults. Given shrinking time horizons for older versus younger adults, which have been associated with different (socio-emotional) life goals^43^, it would be an interesting venue for future studies to differentiate short- and long-term consequences of prosocial behaviors and age differences therein.

It is interesting to note that across many of the measured variables, inconsistencies (and indeed, often times null effects of age) with previously reported age-related results were observed. More specifically, compared to published reports on effects of adult age on prosocial behaviors, well-being, and empathy, we observed null effects of age on prosociality and well-being, and a small quadratic effect on empathy. In the following, we speculate about where these discrepancies may arise from.

First, such discrepancies may reflect different motivational aspects elicited by different study designs (e.g., in-lab vs. real-life, experimental vs. self-reported^81^). On one hand, there is evidence for external validity of commonly used tasks in the domain of prosociality^82,83^. On the other hand, a previous study showed that lab-induced prosocial behavior differed drastically from real-life prosocial behavior in a natural-field dictator game. The authors interpreted these findings as the authors interpreted as inflated prosocial behavior elicited by the lab context^84^. Further, observability has been shown to be associated with higher prosocial behavior^85^, underpinning the assumption that lab-induced prosocial behavior might be different in nature from prosocial behavior in real life. Further, different studies identified differential drivers of empathy. The likelihood to engage in empathy-eliciting situations increased with monetary incentives, and also when the target of empathy was familiar^86^. It has also been found increased perceived closeness leads to better perspective taking abilities in older adults^87,88^, and both, our own age and the social interaction partner’s age affect these capacities^89^.

Second, it has been shown that the sampling strategy influences the degree of prosociality. Indeed, in many age-comparative studies, the younger age group might consist predominantly of students (e.g.,^9,10^), who have been demonstrated to display systematically less prosocial behavior in in-lab studies. This remains true when controlling for age in this population^90^. A strength of the current study is that the sample was quota-sampled on six key demographic variables (i.e., sex, ethnicity, education, geographic region, income, and age), even though representativeness might not be given with respect to other demographics (e.g., marital status, current living situation), as the sample is not random. Moreover, there was a reduced number of participants sampled from the youngest (18–24 years) and oldest (65 years and older) age group, which is why the two youngest and two oldest groups were merged for all analyses. Thus, conclusions about differences with respect to these groups, and comparability with ageing studies, which typically include a wider range of older adults, are limited.

A methodological strength of the dataset analyzed here is the experience-sampling method used to acquire data. EMA studies help to provide closer and deeper insights and to broaden our understanding of real differences across the lifespan by investigating the frequency, intensity, and complexity of different measures. Advantages lie in increased ecological validity, decreased recall bias, and the possibility to measure variability and change over a short time. Despite its many advantages, repeatedly responding to the survey could also have an influence on whether people notice empathy opportunities (for a more detailed discussion of representativeness, potential training, and fatigue-effects see Depow et al.^63^). Experience sampling also shares with other self-report measures its susceptibility to biases, like social desirability. More objective, implicit measures of empathy (e.g., physiological reactions towards others’ suffering) have been used in the lab and have also revealed age differences^11,91^. A limitation of the current study is that we exclusively relied on explicit EMA reports and did not include other, complementary measures of empathy. A recent study^92^ adopted a combined EMA-fMRI approach in young adults to show that affective components of empathy ratings assessed via EMA were associated with behavioral measures from an experimental empathy paradigm, but not with neural activation. Daily cognitive components of empathy ratings assessed using EMA were correlated with neural activation in the medial prefrontal cortex, but not significantly related to perspective taking performance in an experimental task. To gain a more comprehensive insight into the adult lifespan development of the social mind, such multi-modal designs combining EMA, experimental, and physiological measures^93,94^ are a promising venue for future lifespan studies.

## Conclusion

Factors that contribute to, or result from, successful social interactions like empathy, prosocial behavior, and well-being are important throughout life. This analysis of cross-sectional experience-sampling data suggests that most of these constructs show no significant age-related differences when measured repeatedly via self-report in daily life. One exception was empathy. Here we observed a weak inverted U-shaped effect of age on empathy over the course of the lifespan. Future studies should take into account methodological differences stemming from varying study-designs and construct definitions, which might be one source of inconsistencies in age-related results in the literature. Multivariate studies combining standardized in-lab experiments with real-life experience-sampling data are a promising venue for future lifespan studies on the social mind.

## Methods

The current study is based on the publicly available dataset from Depow and colleagues^63^ who used ecological momentary assessment to increase ecological validity and to explore within-person differences. In the current study, we focused on analyzing adult age-related differences on different aspects of daily empathy, daily prosocial behavior, and daily subjective well-being. In the following, we reiterate the methodological approach used by Depow and colleagues^63^.

### Participants

Quota-sampling was used for a nearly representative U.S. sample, in cooperation with the survey company Qualtrics (Qualtrics®, 2002; www.qualtrics.comwww.qualtrics.com). Overall, 3486 participants filled in a demographic questionnaire, including informed consent about participating in a “Daily Interactions’ study”. In a next step, 841 quota-sampled participants were invited via email to participate in the study. Altogether, 375 completed the baseline survey (trait questionnaire measures, instruction to download the app, glossary of the terms), whereas 285 participants completed the full experience sampling for one week, seven times per day. Participants were excluded if they missed more than 7 surveys in total, which resulted in a sample of 246 participants. Three more participants, were excluded from the current analysis due to missing information regarding their age. In the dataset from Depow and colleagues ^63^ age was originally measured in six age groups. The groups were divided into (1) 18 to 24 years (*n* = 14), (2) 25 to 34 years (*n* = 57), (3) 35 to 44 years (*n* = 59), (4) 45 to 54 years (*n* = 51), (5) 55 to 64 years (*n* = 42), and (6) 65 years and older (*n* = 20). Due to comparably small sample sizes in the youngest and oldest age groups Depow and colleagues^63^ merged the two youngest and the two oldest age groups for a more homogenous sample size across the groups, an approach which we adopted for the current analysis. Thus, the final sample consisted of 243 participants (18–34: *n* = 71, 45 female, 3 “other”; 35–44: *n* = 59, 28 females; 45–54: *n* = 51, 34 female; 55 and older: *n* = 62, 29 female). The age groups differed significantly with respect to their gender and education distribution, but not in surveys answered, income, and religiosity (see Table 3 for descriptive and interferential statistics). Participants answered a total of 7141 surveys, which we consider a dataset characterized by a high degree of ecological validity. Due to a bug in the experience sampling procedure in the original study by Depow et al.^63^, a very small number of participants (*n* = 58 out of a total of *n* = 243 participants), received prompts to fill out the survey more often, and sometimes within a shorter time interval (i.e., < 15 min). These cases could be problematic, since the experience sampling was done within a time interval that was shorter than 15 min, which may not have capture participants’ responses to independent events/experiences. Thus, all surveys that were prompted < 15 min after a previous survey were excluded from the analyses (a total of *n* = 110 surveys from a total of *n* = 7251 surveys, corresponding to only 1,5% of all data). Also due to this issue, we were able to include eight surveys per day for a minority of participants (*n* = 18), instead of the intended maximum of seven surveys per day, all of which by time windows ≥ 15 min. For information on how often participants reported an empathy opportunity, having been the target of empathy, and prosocial behavior, as well as reported well-being see Table 4. Further, we observed differences in how often participants reported an empathy opportunity, an opportunity to be the target of empathy, and prosocial behavior, as well as the reported well-being scores (i.e., questions asked on the first level of the survey) as a function of the different survey days. While these differences are mainly driven by the first day of the survey compared to later points in time, they are apparent across all age groups (compare Table 4) and do not differ significantly between the different age groups (all adj. *p*s > 0.236). All participants provided informed consent regarding their participation in the project, and were told they were free to cease their participation at any point. All procedures were approved by the University of Toronto Research Ethics Board to ensure they adhered to relevant ethical guidelines for human data collection and usage (Protocol No. 36941).

**Table 3:** Descriptive and interferential statistics of the final sample characteristics.

	18–34 years	35–44 years	45–54 years	55 + years	Test statistic
Surveys answered	29.46 ± 11.73	29.49 ± 11.12	30.06 ± 12.00	28.65 ± 12.50	*F* = 0.09, *p* = 0.78
Gender (m/f/o)	23/45/3	31/28/0	17/34/0	33/29/0	chi^2^ = 16.46, *p* = 0.012
Education (Highschool/GESD or less/some college/college graduate/ graduate degree)	23/14/20/14/0	12/19/16/8/4	18/11/9/8/5	5/17/20/10/10	chi^2^ = 28.63, *p* = 0.004
Income (under 25,000/25,000–50,000/50,000–100,000/over 100,000)	22/24/21/4	13/23/16/7	18/16/15/2	15/23/16/8	chi^2^ = 7.11, *p* = 0.626
Religiosity (not at all/slightly/religious/strongly/ extremely)	22/17/14/10/5	20/13/15/4/7	11/12/11/12/2	16/17/14/8/5	chi^2^ = 10.40, *p* = 0.581

**Table 4:** Proportion of how often participants reported an empathy opportunity, an opportunity to be the target of empathy, and acting prosocially, as well as the mean value of well-being relative to all surveys answered per day, shown as a function of survey day and age group.

	18–34 years	35–44 years	45–54 years	55 + years
*Cases of Empathy Opportunities (in %)*				
Day 1	37.17	27.79	24.95	35.93
Day 2	25.18	20.15	17.45	21.64
Day 3	19.95	21.52	14.07	23.67
Day 4	23.72	13.62	16.93	23.01
Day 5	20.34	16.91	11.89	18.63
Day 6	15.81	12.74	10.83	16.66
Day 7	16.58	13.32	14.06	15.22
*Cases of Target Opportunities (in %)*				
Day 1	20.09	16.10	15.08	19.88
Day 2	16.06	12.78	8.76	10.95
Day 3	13.06	12.46	11.50	19.63
Day 4	15.63	9.56	11.02	8.25
Day 5	16.64	8.46	9.68	9.16
Day 6	11.80	10.43	8.19	13.08
Day 7	13.56	6.11	9.02	13.78
*Cases of Prosocial Behavior (in %)*				
Day 1	38.94	27.39	29.56	35.41
Day 2	29.19	22.63	20.65	21.98
Day 3	22.43	22.47	16.63	20.87
Day 4	19.96	17.88	14.31	23.92
Day 5	17.72	18.54	13.13	20.43
Day 6	21.08	14.10	10.62	19.52
Day 7	21.24	15.16	14.40	17.69
*Well-Being (Mean)*				
Day 1	4.94	5.09	4.98	5.24
Day 2	4.99	4.92	5.04	5.34
Day 3	5.05	5.00	4.95	5.23
Day 4	4.91	5.03	4.97	5.16
Day 5	4.84	4.92	4.90	5.23
Day 6	4.79	4.95	5.13	5.26
Day 7	4.89	5.28	5.19	5.11

### Procedure

#### Baseline survey

Participants first underwent a baseline survey to collect demographic information and trait measures (compare^63^). The baseline survey included a glossary of the important terms (empathy opportunity, emotion and sharing, perspective taking, compassion, prosocial behavior), to ensure that all participants would understand the concepts in the same way. For detailed information about the materials see Depow and colleagues^63^.

#### Experience-sampling survey

After the baseline survey, participants underwent seven short surveys per day, sent between 10am and 10 pm for one week, delivered by Metricwire (MetricWire®, 2013). Surveys were sent semi randomly, within a 90-min window, with a minimum 15 min gap between surveys. Surveys expired 20 min after the prompt. The daily survey consisted of four levels that were built on each other (see Fig. 1). On the first level, participants were questioned about their current subjective well-being, and if they had an opportunity to empathize (empathy opportunity), to be the target of empathy, or to act prosocially in the last 15 min. If an empathy opportunity had occured in the last 15 min, further details relating to this opportunity were acquired. On the second level, participants were asked if they experienced actual feelings of empathy for the person or people involved. If they responded “yes”, they were subsequently asked to indicate whether they experienced any (or several) of emapthy's subcategories: emotion sharing, perspective taking, and compassion. Subsequently, extent, difficulty, and confidence were probed for every subcategory they indicated. The length of the survey was always the same, irrespective of the answer provided. In the original study, a multitude of questions were asked, which are not relevant for the current research question. For further details see Depow and colleagues^63^.

### Statistical analysis

All data were analyzed using R (Version 4.0.3)^95^ with RStudio^96^. We adopted the statistical approach described in Depow and colleagues^63^ but, additionally examined the effect of age group and interactions with age group on our variables of interest. Age group entered all models as a continuous variable, based on the assumption of ordinality and continuity (e.g.,^11,64^). Age differences in sample characteristics (compare Table 3) were examined with Chi^2^-tests for categorical variables and ANOVA for continuous variables using the R package “stats”^95^ and “rstatix”^97^.

Age-differences regarding the different aspects of daily empathy, prosocial behavior, and well-being were analyzed with mixed-effect models, using the *mixed* function from the “afex” package^98^. Binary outcomes (experience of empathy (yes/no), engaging in prosocial behavior (yes/no)) were analyzed with generalized mixed-effects models and p-values were calculated with likelihood-ratio tests. Well-being as a continuous outcome (global well-being score resulting from happiness and sense of purpose) was analyzed by using mixed-effects models, with p-values calculated based on the Satterthwaite method^98^. For each outcome variable, we constructed two models as follows: i) including age as a linear predictor ii) including age both, as a linear and a quadratic predictor. Model selection was conducted based on a loglikelihood ratio test (R function *anova)*. In the results section, the results of the better fitting model are reported, respectively. All models were nested within participant and survey day as random intercepts. In case of convergence warnings, the number of iterations were increased and the optimizer changed to “bobyqa”, followed by reducing the maximum random effect structure by survey day. We controlled for gender, income, religiosity, and education by including them as covariates, each in a separate model. Controlling for these four different covariates did not change the significance level of our predictors of main interest (i.e., age). Thus, in the results section only the models without covariates are reported.

In order to analyze between- and within-subject effects regarding the influence of empathy and age on prosocial behavior and well-being, and prosocial behavior and age on subjective well-being, predictors were centered in different ways. Continuous variables were participant-centered for within-subject effects, and grand-mean centered for between-subject effects. Binary variables were dummy-coded (1 = yes, 0 = no) for within-subject effects and for between-subject effects the grand-mean centered average proportion of yes responses of a participant was used (for further details see^63^). Model statistics, including effect sizes “r” for fixed effects in mixed-effect models derived from R^2^^99^ were calculated with the *summaryh* function from the “hausekeep” package^100^. The *p-adjust* function from the “stats” package^95^ was used to correct p-values for multiple testing with the false discovery procedure^101^. Data are publicly available at: https://osf.io/y3ud7/, and scripts are publicly available at: https://osf.io/fdmtg/.

## Supplementary Information

Supplementary Information.

## Data Availability

All data and materials are publicly available at: https://osf.io/y3ud7/, and scripts are publicly available at: https://osf.io/fdmtg/.
