# Publisher Correction: Investigating adult age differences in real-life empathy, prosociality, and well-being using experience sampling

**DOI:** 10.1038/s41598-023-40699-0

**Published:** 2023-08-23

**Authors:** Lena Pollerhoff, Julia Stietz, Gregory John Depow, Michael Inzlicht, Philipp Kanske, Shu‑Chen Li, Andrea M. F. Reiter

**Affiliations:** 1 Lifespan Developmental Neuroscience, Faculty of Psychology, Technische Universität Dresden, Dresden, Germany; 2 Clinical Psychology and Behavioral Neuroscience, Faculty of Psychology, Technische Universität Dresden, Dresden, Germany; 3 Department of Psychology, University of Toronto, Toronto, ON Canada; 4 Rotman School of Management, University of Toronto, Toronto, ON Canada; 5 Max Planck Institute for Human Cognitive and Brain Sciences, Leipzig, Germany; 6 Centre for Tactile Internet with Human-in-the-Loop, Technische Universität Dresden, Dresden, Germany; 7 Department of Child and Adolescent Psychiatry, Psychosomatics and Psychotherapy, University Hospital Würzburg, Würzburg, Germany; 8 German Centre of Prevention Research On Mental Health, Julius-Maximilians-Universität Würzburg, Würzburg, Germany

Correction to: *Scientific Reports* 10.1038/s41598-022-06620-x, published online 02 March 2022

The original version of this Article contained errors, where Table 4 was incomplete. Furthermore, the statement under the column “55+” in the row “Day 1” was incorrectly placed into Table 4. The original Table 4 and accompanying legend appear below.

**Table 4:** Proportion of how often participants reported an empathy opportunity, an opportunity to be the target of empathy, and acting prosocially, as well as the mean value of well-being relative to all surveys answered per day, shown as a function of survey day and age group.

	18–34 years	35–44 years	45–54 years	55+ years
*Cases of empathy opportunities (in %)*				
Day 1	37.17	27.79	24.95	All participants provided informed consent regarding their participation in the project, and were told they were free to cease their participation at any point. All procedures were approved by the University of Toronto Research Ethics Board to ensure they adhered to relevant ethical guidelines for human data collection and usage (Protocol No. 36941)

“All participants provided informed consent regarding their participation in the project, and were told they were free to cease their participation at any point. All procedures were approved by the University of Toronto Research Ethics Board to ensure they adhered to relevant ethical guidelines for human data collection and usage (Protocol No. 36941).” has now been placed correctly in the Methods section, under the sub-section ‘Participants’.

The original Article has been corrected.

