# Supplementary material for: Investigating adult age differences in real-life empathy, prosociality, and well-being using experience sampling
Source: Sci Rep. 2022 Mar 2;12:3450. doi: 10.1038/s41598-022-06620-x (PMC8891267; doi:10.1038/s41598-022-06620-x)

**Supplementary Information**

**Supplementary table S1.** Age predicting extent, difficulty, and confidence of the three subcomponents emotion share, perspective take, and compassion. Statistics obtained from mixed models, nested within participant and survey day. Each interaction ran in a separate model. P-values were adjusted to control the false discovery rate. * p < .05, ** p < 0.01. ^α^ Fixed effect structure of the model include religiosity as covariate.

|  | ***t* or *z*-score** | ***p*-value** | **Adj. *p*-value** | **Estimate (*SE*)** | **Effect size (*r*)** |
| --- | --- | --- | --- | --- | --- |
| Emotion share extent ~ age | -0.50 | .620 | .791 | 0.06 | 0.04 |
| Emotion share difficulty ~ age | -1.13 | .261 | .587 | 0.07 | 0.08 |
| Emotion share confidence ~ age | -0.39 | .695 | .791 | 0.05 | 0.03 |
| Perspective take extent ~ age ^α^ | -1.96 | .051 | .230 | 0.06 | 0.16 |
| Perspective take difficulty ~ age | -1.16 | .249 | .587 | 0.08 | 0.09 |
| Perspective take confidence ~ age | 0.38 | .703 | .791 | 0.05 | 0.03 |
| Compassion extent ~ age | -0.07 | .942 | .942 | 0.05 | 0.01 |
| Compassion difficulty ~ age | -2.63 | .009** | .081 | 0.07 | 0.20 |
| Compassion confidence ~ age | 0.96 | .337 | .607 | 0.04 | 0.08 |

**Supplementary Figure S1.** Diagram regarding the co-occurrence of the subcomponents emotion share, perspective take, and compassion in younger adults (18-34 years old). Each circle reflecting the relative percentage of the three subcomponents, both occurring individually or concurrently, when actual feelings of empathy were reported. The large circle in the middle demonstrates the percentage of co-occurrence with respect to all three subcomponents, whereas the circles in the light gray ring show the co-occurrence of only two subcomponents.


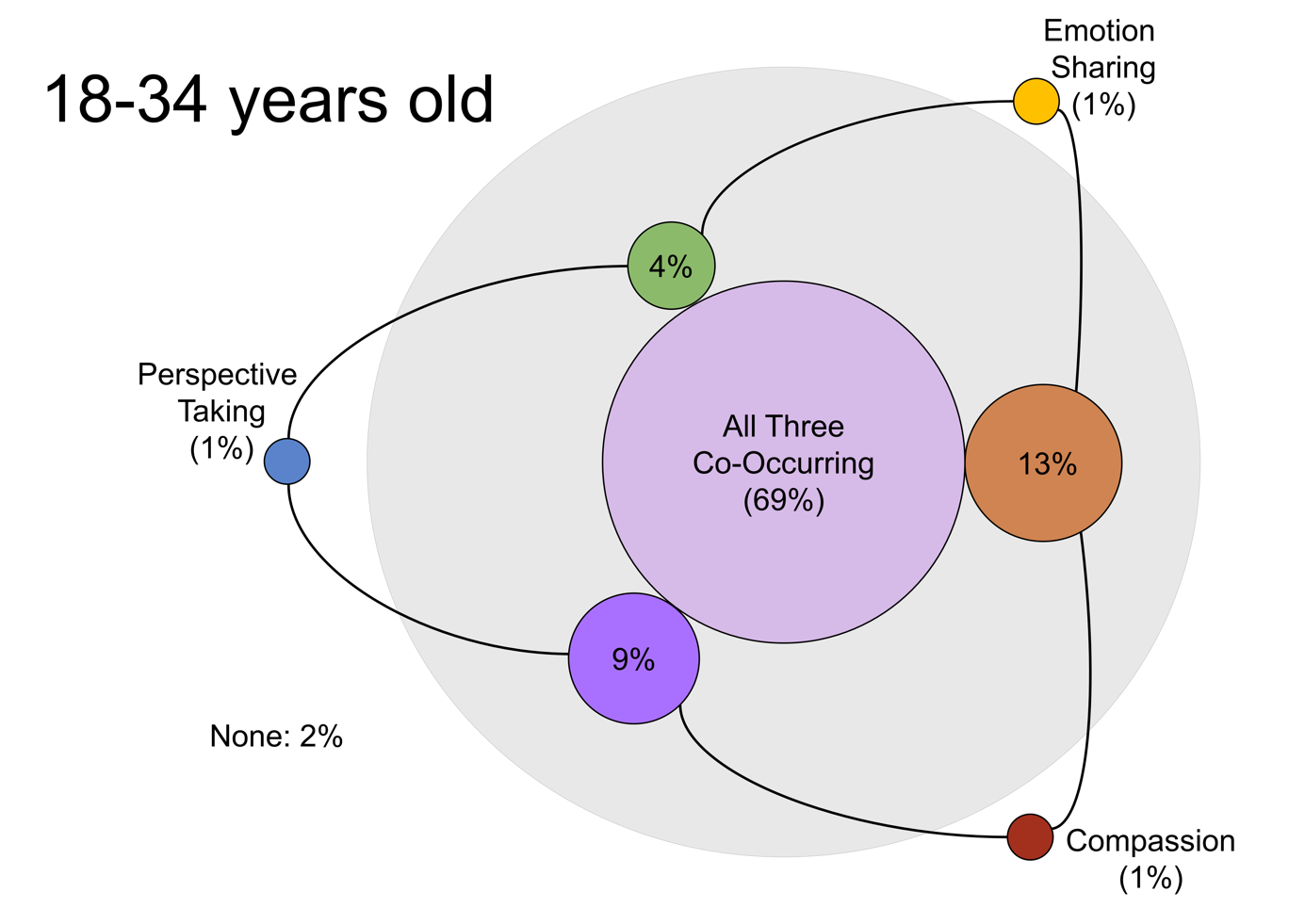


**Supplementary Figure S2.** Diagram chart regarding the co-occurrence of the subcomponents emotion share, perspective take, and compassion in middle age adults (35-44 years old). Each circle reflecting the relative percentage of the three subcomponents, both occurring individually or concurrently, when actual feelings of empathy were reported. The large circle in the middle demonstrates the percentage of co-occurrence with respect to all three subcomponents, whereas the circles in the light gray ring show the co-occurrence of only two subcomponents.


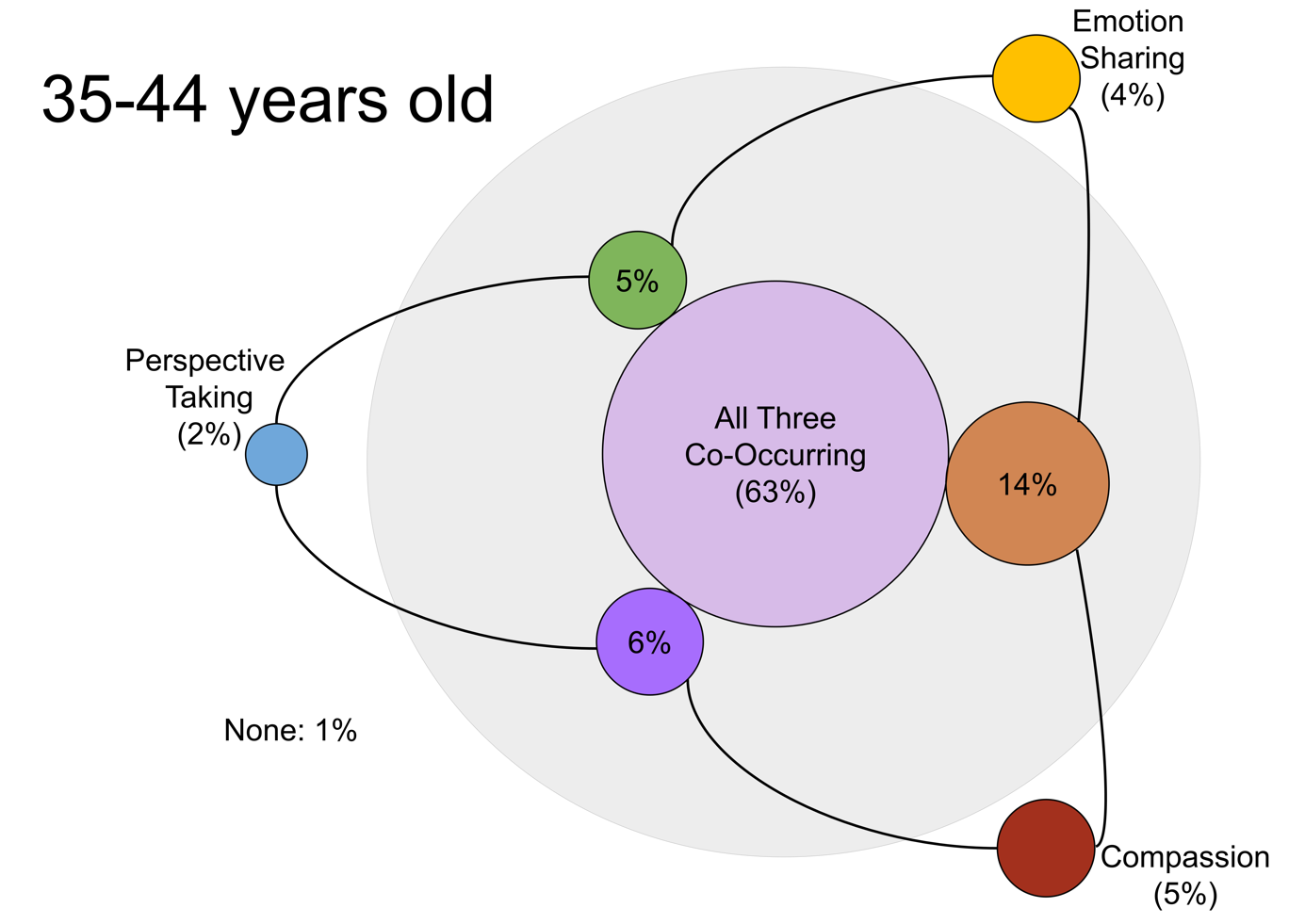


**Supplementary Figure S3.** Diagram regarding the co-occurrence of the subcomponents emotion share, perspective take, and compassion in middle age adults (45-54 years old). Each circle reflecting the relative percentage of the three subcomponents, both occurring individually or concurrently, when actual feelings of empathy were reported. The large circle in the middle demonstrates the percentage of co-occurrence with respect to all three subcomponents, whereas the circles in the light gray ring show the co-occurrence of only two subcomponents.


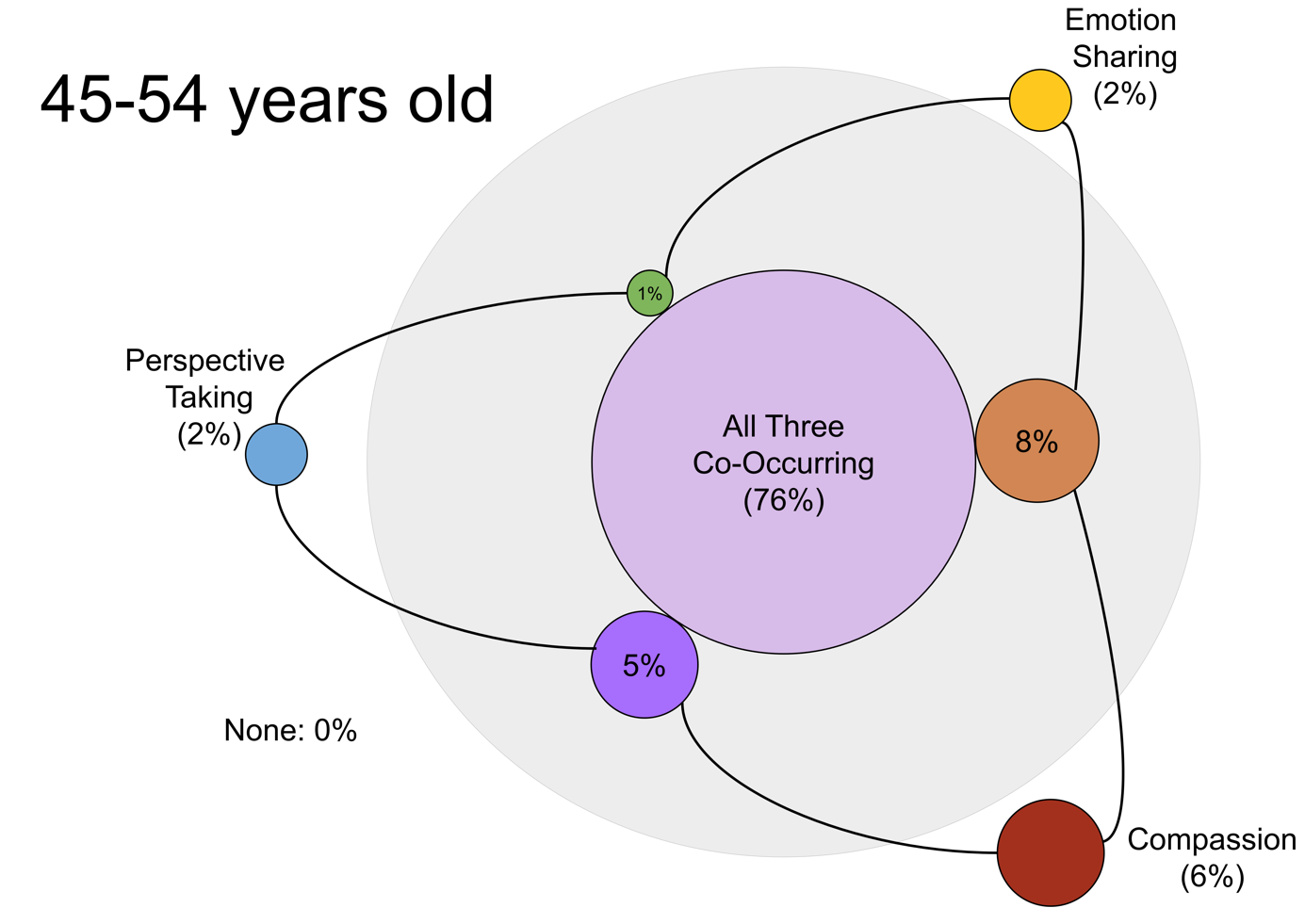


**Supplementary Figure S4.** Diagram regarding the co-occurrence of the subcomponents emotion share, perspective take, and compassion in old adults (55 years and older). Each circle reflecting the relative percentage of the three subcomponents, both occurring individually or concurrently, when actual feelings of empathy were reported. The large circle in the middle demonstrates the percentage of co-occurrence with respect to all three subcomponents, whereas the circles in the light gray ring show the co-occurrence of only two subcomponents.


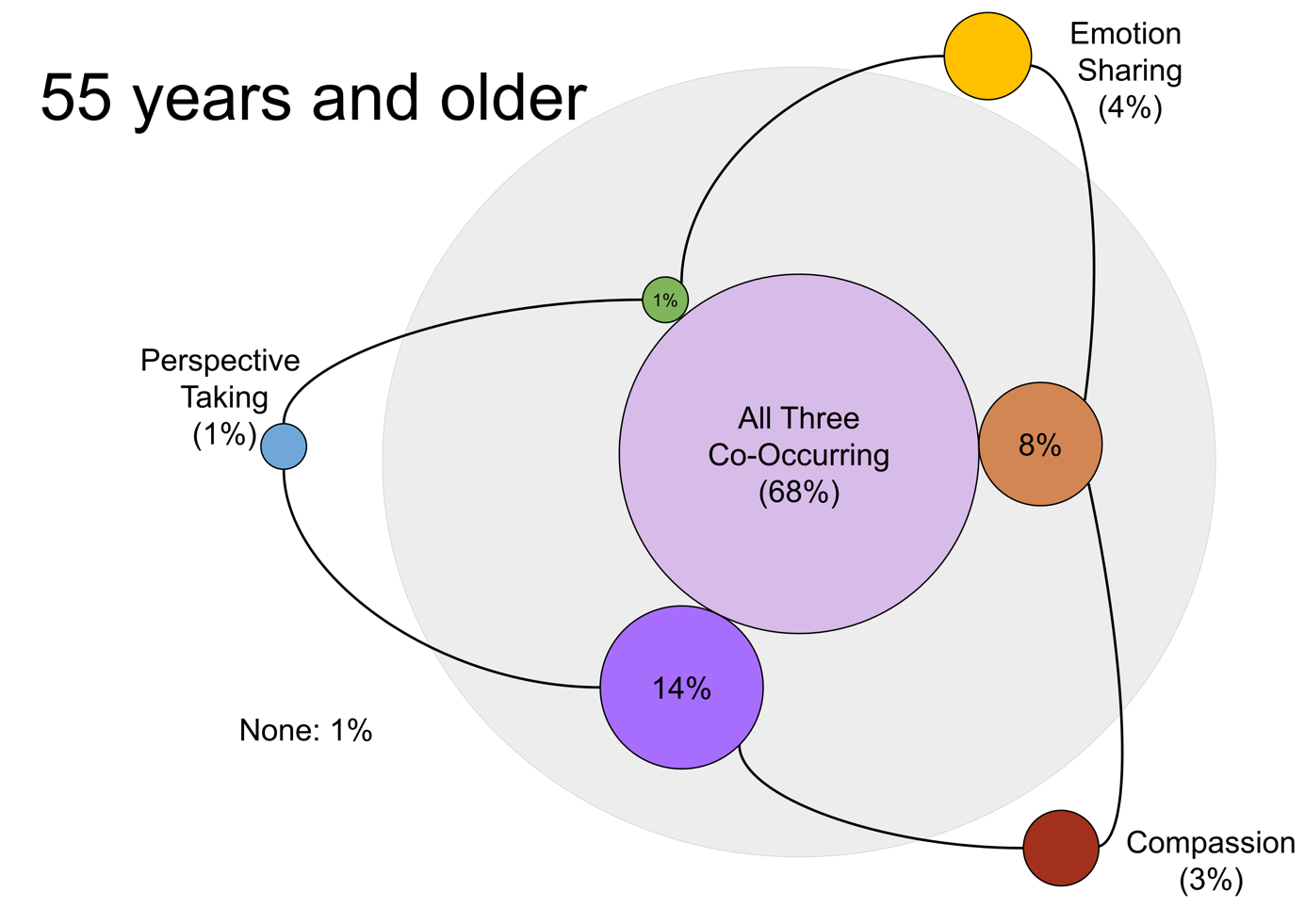

Supplement: Supplementary file 1 — Supplementary Information. [file 41598_2022_6620_MOESM1_ESM.docx]
